# Enhanced Viability of Probiotics against Gastric Acid by One-Step Coating Process with Poly-L-Lysine: In Vitro and In Vivo Evaluation

**DOI:** 10.3390/pharmaceutics12070662

**Published:** 2020-07-14

**Authors:** Shwe Phyu Hlaing, Jihyun Kim, Juho Lee, Dongmin Kwak, Hyunwoo Kim, Jin-Wook Yoo

**Affiliations:** 1 College of Pharmacy, Pusan National University, Busan 46241, Korea; shwephyuhlaing@gmail.com (S.P.H.); jihyunjoshkim@gmail.com (J.K.); jhlee2350@gmail.com (J.L.); kdm318@naver.com (D.K.); rlagusdn0628@naver.com (H.K.); 2 Department of Cogno-Mechatronics Engineering, College of Nanoscience and Nanotechnology, Pusan National University, Busan 46241, Korea

**Keywords:** *Lactobacillus plantarum*, poly-L-lysine, probiotics, acid resistance, viability

## Abstract

Due to their low acid tolerance, a majority of probiotics face difficulties with regard to surviving in the gastric environment long enough to reach the intestinal surfaces where they colonize and provide health benefits. We prepared a probiotic delivery system that can enhance their viability in acidic conditions by developing a one-step poly-L-lysine (PLL) coating process. We determined whether the coating process was successful by measuring the zeta potential and observing it with confocal scanning microscopy. PLL-coated *L. plantarum* (PLL-LP), incubated in a solution of pH 2 for 2 h, exhibited a higher viability (6.86 ± 0.12 log CFU/mL of viable cells) than non-coated *L. plantarum* (non-coated LP), which exhibited only 2.7 ± 1.23 log CFU/mL of viable cells. In addition, a higher amount of *L. plantarum* was detected in the feces of mice orally administered PLL-LP (6.2 ± 0.4 log CFU/g of feces) than in the feces of the control groups. In addition to enhancing probiotic viability in pH 2 solution, the PLL coating showed no effect on the probiotic growth pattern and the viability of either freeze-dried *L. plantarum* or *L. plantarum,* stored at −20 °C and 4 °C, respectively. Overall, these results indicated that the PLL coating is a promising potential probiotic delivery system.

## 1. Introduction

Since 2002, probiotics have been defined by the Food and Agriculture Organization and World Health Organization as “live microorganisms (bacteria or yeast) which when administered in adequate amounts confer health benefits to the host” [1]. Recent studies have shown that probiotic bacteria have beneficial effects not only on the digestive system, but also on other biological systems in humans. It has been established that the bacterial composition in the human gastrointestinal tract (GIT) plays an essential role in the progression of a number of disorders, including obesity, diabetes and cancer [2,3,4,5].

The oral administration of probiotics products has been widely used to relieve specific symptoms and conditions of the GIT (i.e., diarrhea and the imbalance of intestinal microbiota) [6]. Probiotics, as living organisms, encounter challenges to their viability along their path through the GIT. In particular, their susceptibility to pH leads to their low viability in the highly acidic gastric fluid, which results in fewer viable probiotic cells, and therefore a reduced probiotic efficacy and functionality in the GIT [7]. Therefore, developing a successful probiotic delivery system that can protect and enhance the viability of probiotics in the highly acidic gastric fluid is a critical issue.

Several forms of technology have been developed to address these difficulties. Typically, microencapsulation with various biomaterials is used to protect probiotics from direct contact with the external gastric environment, leading to an improved probiotic survival rate [8]. Despite the benefits, there is a concern that, due to the incomplete disintegration of the encapsulating matrix, microencapsulation inhibits direct contact between the probiotics and intestinal surfaces, which is essential for conferring the beneficial properties of the probiotics to the host [9]. To circumvent this problem, layer-by-layer (LbL) polyelectrolyte coatings were developed to coat probiotics without requiring their release from the encapsulating matrix. The mechanism of the electrostatic interaction between the negatively charged membrane of the probiotics and the cationic polymer forms a coating layer on the surface of the probiotic cells. Previous studies of living cell encapsulation with the LbL method have used two oppositely charged (cationic and anionic) polymers and a repeated coating process to obtain the desired stability of the probiotics in acid. The advantages of LbL coatings are conferred by the minimal quantity of polymer used and the direct adherence to the intestinal surface. The drawbacks of multilayer coatings are their time-consuming and tedious production process, and their prevention of the entry of nutrients required for probiotic cell survival, growth, and proliferation. Furthermore, the cells have to break off from the coating layers to grow in size before dividing and multiplying, which results in a longer lag phase prior to the exponential phase [2,10].

To overcome these drawbacks, we hypothesized that coating the surface of the probiotics directly with a cationic polymer by using a one-step coating process could be a promising method for developing a probiotic delivery system that enhances probiotic viability in gastric acid by electrostatic repulsion with H^+^ ion. Although a number of positively charged natural and synthetic polymers are used in drug delivery systems, only a few can be used for probiotic delivery systems because of the fragile viability of probiotics during the manufacturing process. For example, chitosan, the most widely used cationic polymer in probiotic microencapsulation systems, has an antibacterial activity, and therefore cannot be directly coated onto the surface of the probiotics without experiencing a decrease in its cationic charge. In the present study, we coated the surface of the probiotic bacteria *Lactobacillus plantarum* with poly-L-lysine (PLL), which was first identified in the culture filtrate of an actinomycete, *Streptomyces albulus* [11]. PLL is a biocompatible and biodegradable homopolymer of essential amino acid L-lysine [12,13]. It was revealed that PLL is non-toxic, in an acute oral toxicity study in rats, with a dose of up to 5 g/kg [14]. Previously, PLL was used to coat the alginate beads in which probiotics are encapsulated for enhanced viability [15,16]. To the best of our knowledge, however, no studies have used PLL to directly coat probiotic surfaces. *L. plantarum* is often isolated from various pickle preparations, and is widely used as a dietary supplement [17,18,19]. It has been used as a probiotic strain because it is generally regarded as safe (GRAS), and has been reported to have antioxidant and anti-inflammatory properties, strengthen the gut barrier, and have beneficial effects against colitis [17,20,21]. As such, it could be applied in our future studies. In this study, the PLL coating on *L. plantarum* was characterized by zeta potential measurement and confocal scanning microscopy, and the effect of PLL on *L. plantarum* was investigated by analyzing the growth pattern, freeze-drying stability and storage stability of the probiotic. In addition, the in vitro viability loss of poly-L-lysine-coated *L. plantarum* (PLL-LP) against the acidic pH of the gastric environment, and in vivo viability, were investigated in imprinted control region (ICR) mice using non-coated *L. plantarum* (non-coated LP) as a control.

## 2. Materials and Methods

### 2.1. Materials

Poly-L-lysine (PLL) (average molecular weight: 4700) was kindly provided by JNC corporation (Tokyo, Japan) as a gift. Sodium chloride and L-cysteine were purchased from Sigma-Aldrich (St. Louis, MO, USA). Bacto^TM^ Lactobacilli MRS Broth (MRS) and agar were purchased from BD biosciences (San Jose, CA, USA). Bromocresol purple, ciprofloxacin and LIVE/DEAD^TM^ Baclight^TM^ Bacterial Viability Kit were purchased from Thermo Fisher Scientific Inc. (Waltham, MA, USA). Flamma^®^ 648 NHS ester was purchased from BioActs (Incheon, Korea). All other materials and solvents used were of the highest analytical grade.

### 2.2. Microorganisms

Pure culture of *L. plantarum* (KCTC 3108) was obtained from the Korean Collection for Type Cultures. The stock culture of *L. plantarum* was stored at −80 °C in MRS broth with 15% glycerol and activated by spreading onto an MRS agar plate containing L-cysteine. Then, it was incubated at 37 °C for 48 h under anaerobic conditions. A primary starting culture was prepared by transferring one colony from the incubated MRS agar plate to fresh MRS broth with L-cysteine and incubating it for 12 h. The working culture was then prepared with a 1:50 ratio of starting culture to fresh MRS broth and incubated at 37 °C for 24 h in a shaking incubator. The final concentration of *L. plantarum* in the working culture was approximately 1 × 10^10^ log CFU/mL. Before starting the coating process, the working culture was centrifuged at 2500 g for 10 min at 4 °C. The supernatant was discarded, and the pellet was washed three times with normal saline.

### 2.3. Enumeration of Viable Cells

In this study, *L. plantarum* viability was quantified by using the plate counting method with MRS agar [22]. In detail, after serial dilutions of the cells, 100 μL of diluted solution was spread onto each MRS agar plate and incubated at 37 °C for 48 h in anaerobic conditions using an anaerobe container system (BD GasPack^TM^ EZ, Sparks, NE, USA) according to the manufacturer’s instructions. All samples were independently produced in triplicates, and viable cells were counted in terms of colony-forming units (CFU) on the MRS agar plates.

### 2.4. Poly-L-Lysine (PLL) Coating onto the Surface of L. Plantarum

**Scheme 1:**
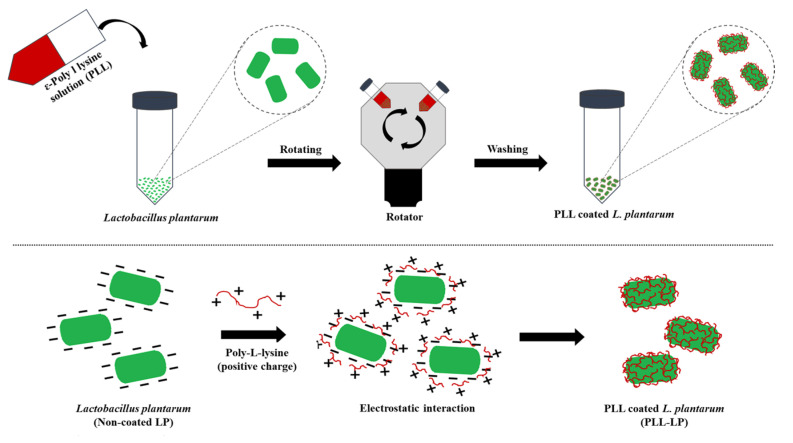
Schematic illustration of coating poly-L-lysine onto the probiotic cell surface, and of the electrostatic interaction between the negatively charged surface of *L. plantarum* and the positively charged PLL.

As shown in Scheme 1, freshly harvested and washed *L. plantarum* were re-suspended in a sterilized PLL solution in which the pH was adjusted to 6 based on the pKa value of PLL, to protonate the PLL amino group. As a probiotic is a living cell, the selection of solvent, pH, temperature and concentration were carefully determined. After optimizing the method, the coating was applied by gently rotating the cells for 15 min. After rotation, the probiotic cells were pelletized by centrifugation at 2500 g for 10 min at 4 °C, and excess PLL solution was removed by washing three times with 0.15 M NaCl solution. The same rotation time and washing steps were performed with uncoated cells in 0.15 M NaCl solution as a control.

### 2.5. Characterization of the PLL Coating on L. Plantarum

#### 2.5.1. Zeta Potential

Successful coating of the negatively charged *L. plantarum* surface with the positively charged polyelectrolyte PLL was characterized by determining the zeta potential with a Malvern Zeta analyzer, Nano-ZS90 (Malvern, UK). The zeta potential was measured using double-distilled water as a dispersing medium. Each data point was obtained by taking the average of five independent measurements and their standard deviations.

#### 2.5.2. Confocal Scanning Laser Microscopy

The coating of PLL on the surface of the *L. plantarum* was observed using a ZEISS LSM 800 confocal microscope (Carl Zeiss, Oberkochen, Germany) equipped with 63× magnification. First, freshly prepared non-coated LP and PLL-LP were each interacted with an equal amount of Flamma^®^ 648 NHS ester dye for 1 h in the dark. Flamma^®^ 648 NHS ester dye interacts with primary amine groups and forms strong amide bonds. As PLL is a homo-polypeptide of a lysine residue, it is rich in primary amine groups; therefore, a red signal indicates the presence of PLL. After their interaction with the NHS ester dye, free dye was removed by centrifugation at 2500 g for 10 min at 4°C three times with double-distilled water. Then, the pelleted cells were stained with SYTO 9 dye according to the manufacturer’s protocol, to observe the shape of the probiotics. The suspended cells were fixed on a glass slide and observed with a confocal laser microscope using excitation/emission wavelengths of 648/663 nm and 480/500 nm, to observe the fluorescence of the NHS ester and that of the SYTO 9, respectively.

### 2.6. Growth Pattern

The growth pattern of PLL-LP was determined, in order to evaluate the effect of the PLL coating on the growth of the cells. Both non-coated LP and PLL-LP were cultured anaerobically in MRS broth by shaking at 220 rpm in a shaking incubator at 37 °C. The turbidity of the bacterial solution, which indicates the increase in the growth of bacteria, was determined by measuring the optical density (OD) at a wavelength of 600 nm [23]. The turbidity of the MRS growth media broth was measured at predetermined time points until the stationary phase was reached. The degree of turbidity at the wavelength of 600 nm was measured by UV-Vis spectrophotometry (HITACHI U-5100 spectrophotometer, Tokyo, Japan). All results were obtained in independent triplicates.

### 2.7. In Vitro Survival of Non-Coated LP and Poly-L-Lysine-Coated L. Plantarum (PLL-LP) Against Gastric Acid

In this study, the viability of non-coated LP and PLL-LP in an acidic solution was determined. Specifically, a low-pH solution (pH 2) was prepared by using a 0.2% sodium chloride solution with diluted hydrochloric acid to simulate the highly acidic human stomach conditions. Both non-coated LP and PLL-LP were incubated in prepared pH 2 solution for 2 h. To evaluate the exact number (in log CFU) of viable non-coated LP and PLL-LP cells, the incubated probiotics suspended in gastric pH for 1 and 2 h were serially diluted and 100 μL of each dilution was spread onto MRS agar plates. The agar plates were incubated at 37 °C in anaerobic conditions for 48 h, and then the number of colonies on the agar plates was counted to confirm bacterial viability. Each data point was obtained by taking the average of eight independent measurements and their standard deviations. Additionally, after 2 h of incubation in an acidic solution, the probiotic cells were stained with the LIVE/DEAD^TM^ Baclight^TM^ Bacterial Viability Kit according to the manufacturer’s protocol. The samples were then observed with a ZEISS LSM800 confocal laser microscope with 20× magnification and excitation/emission maximum wavelengths of 480/500 nm and 490/635 nm, to observe the SYTO 9’s and the propidium iodide’s (PI) fluorescence, respectively.

### 2.8. Storage and Freeze-Drying

To confer the desired health benefits, a sufficient number of probiotics must survive not only the gastric conditions but also the final manufacturing processes [24]. Viable probiotics are recommended to be present at a minimum level of 7 log CFU/g in a fermented food product to confer health benefits [25]. To determine whether the PLL coating influenced the storage and freeze-drying stability of *L. plantarum*, the viability of non-coated LP and PLL-LP without added cell protectant was evaluated during the storage and freeze-drying processes. To determine storage stability at 4 °C, equal amounts of freshly prepared and pelletized non-coated LP and PLL-LP were put in separate storage tubes and stored at 4 °C in a refrigerator for 4 weeks. We also determined the protective effect of PLL against the freeze-drying process. Pelletized probiotic cells were first kept at −80 °C for 7 h and then subsequently lyophilized in a freeze-drier overnight. The freeze-dried samples were kept in a −20 °C refrigerator to evaluate their stability. The viability of the storage samples (at 4 °C and −20 °C) was evaluated weekly for 4 weeks.

### 2.9. In Vivo Viability Test

Animal experiments were performed in accordance with the regulations of Pusan National University Institutional Animal Care and Use Committee (PNU-IACUC) (approval date: 17 April 2020; approval Number: PNU-2020-2589). Male ICR mice (7-week old; weighing 30–35 g) were chosen as the animal model. Animals were provided food and water and kept at 25 °C ± 3 °C in a 12-h light/dark cycle. Animals were divided into 3 groups of 4 mice each: the control (distilled water), non-coated LP, and PLL-LP groups. Mice in the LP and PLL-LP groups were administered 2 × 10^8^ log CFU/mL of cells in distilled water by oral gavage. After 24 h, mice were housed individually, and feces was collected within 10 min of defecation. Each feces sample was put in a sterilized conical tube, weighed, and serially diluted with sterilized normal saline solution for the enumeration of viable cells. To avoid contamination with external microorganisms, all processes were operated with a sterilized apparatus and solvent. To avoid detecting other bacteria in the mouse feces, a selective agar plate for *L. plantarum* [26,27] was used. As in the former test, the agar plates of culture spread were incubated anaerobically for 48 h at 37 °C. Mice in the control group were administered distilled water by oral gavage.

### 2.10. Statistical Analysis

Statistical analyses of all in vitro and in vivo data were performed using two-way ANOVA and one-way ANOVA followed by the Bonferroni test in GraphPad Prism 5.0 (GraphPad Software, Inc., LA Jolla, CA, USA). *P*-values less than 0.05 were considered statistically significant.

## 3. Results and Discussion

### 3.1. Coating of PLL on L. Plantarum

Unlike previous LbL technical methods and materials used for probiotic encapsulation systems, we used only one biopolymer to form a single-layer coating for the probiotic cells. We used the well-known mechanism of electrostatic adsorption to coat polycationic PLL onto the negatively charged cell surface of *L. plantarum*. Various concentrations of PLL (0, 0.5, 1, 1.5 and 2 mg/mL) were coated onto the probiotics, and the zeta potential was measured. The zeta potential of *L. plantarum* changed from negative to positive (mV) when the coating concentration of PLL reached 1 mg/mL. PLL coating concentrations higher than 1.5 mg/mL produced no significant change in the zeta potential. This may be due to the limited surface area of *L. plantarum*; the excess PLL would have been removed by the washing process. In this study, we chose to use 1.5 mg/mL of PLL for further experimentation, as this concentration produced the highest positive charge.

**Figure 1:**
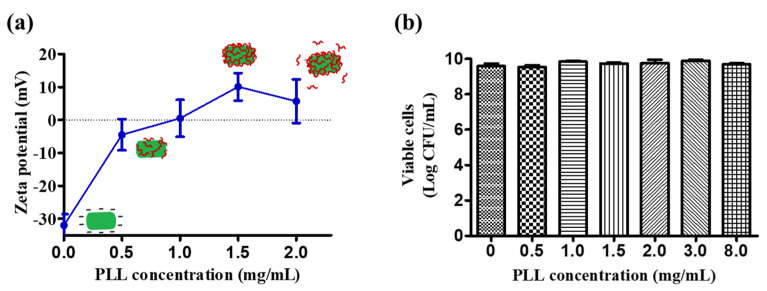
Optimization of the PLL coating on the surface of *Lactobacillus plantarum.* (**a**) Zeta potential of *L. plantarum* after coating with different concentrations of the PLL solution. (**b**) Viability of *L. plantarum* after incubation with different concentrations of the PLL solution for 15 min. The error bar represents the standard deviation (*n* = 5).

As PLL is a cationic polymer, there was a concern that the *L. plantarum* membrane would be destroyed due to the positive charge. Most of the cationic polymers widely used in drug delivery systems, such as chitosan and polyethyleneimines, also present this challenge because of their antimicrobial activity. Although PLL has been shown to inhibit the growth of microbial agents, the minimum inhibitory concentration (MIC) depends on the strain of bacteria, the pH, and the incubation time intervals [11]. To determine the safety of the PLL coating, the viability of the *L. plantarum* was evaluated after it was coated with different concentrations of PLL. As shown in Figure 1b, concentrations of PLL up to 8 mg/mL did not have significantly negative effects on the viability of *L. plantarum* during the 15-min incubation period. The viability of *L. plantarum* did not show any significant changes after being coated with a 1.5-mg/mL PLL solution (9.6 ± 0.1 log CFU/mL before coating, and 9.7 ± 0.1 log CFU/mL after). According to this result, the process of coating with a 1.5-mg/mL PLL solution did not affect the viability of *L. plantarum*.

### 3.2. Characterization of the Poly-L-Lysine Coating on L. Plantarum

**Figure 2:**
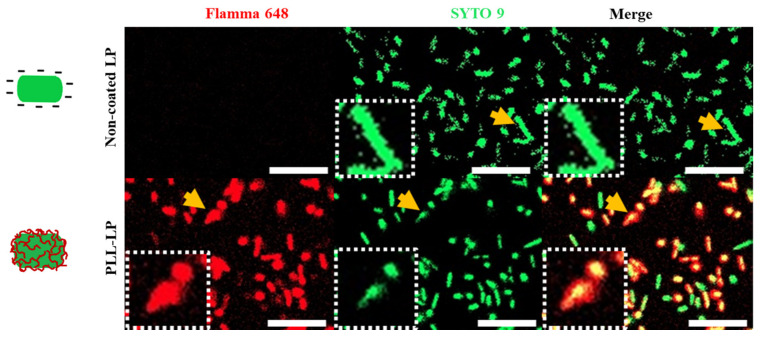
Confocal laser scanning micrographs of non-coated LP and PLL-LP stained with Flamma^®^ 648 NHS ester fluorescence dye (red signal) and SYTO 9 (green signal) for conjugation with the -NH_2_ group of PLL and for the shape of the probiotics cells, respectively. The yellow arrows indicate the magnified *L. plantarum* in the white-dotted squares. The scale bar represents 10 µm.

After optimizing the concentration and incubation time, *L. plantarum* was coated with a 1.5-mg/mL PLL solution, as shown in Scheme 1. The successful coating on the surface of the *L. plantarum* with PLL was determined by staining with two fluorescents dyes. A red signal, indicating the presence of PLL, was observed throughout the PLL-LP, whereas no red fluorescence was observed in the non-coated LP, as shown in Figure 2. Conversely, the nucleic acid stain SYTO 9, which can enter cells and bind to nucleic acids, produced a green fluorescence signal in both the non-coated LP and PLL-LP that is used to visualize the shape of the probiotics’ cells. According to this result, we confirmed the presence of PLL on the surface of the PLL-LP.

### 3.3. Growth Pattern

**Figure 3:**
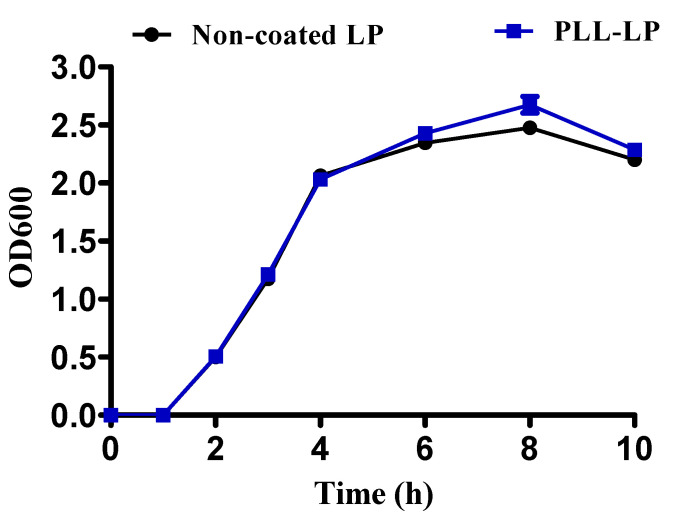
Growth pattern of non-coated *L. plantarum* (non-coated LP) and PLL-coated *L. plantarum* (PLL-LP) incubated in MRS broth for 10 h at 37 °C. The error bar represents the standard deviation (*n* = 3).

In our study, the single-layer PLL coating on *L. plantarum* did not influence the growth pattern of the probiotic in the presence of MRS medium at 37 °C in a shaking incubator. As shown in Figure 3, within 2 h of incubation, the exponential phases of both non-coated LP and PLL-LP began. The OD600 values of both non-coated LP and PLL-LP gradually increased along with the number of probiotic cells, until the stationary phase was reached within 10 h of incubation in the shaking incubator. Consequently, we found that the PLL coating layer does not inhibit growth and proliferation of the probiotic cells.

### 3.4. In Vitro Survival of Non-Coated and PLL-Coated L. Plantarum in Gastric Acid

**Figure 4:**
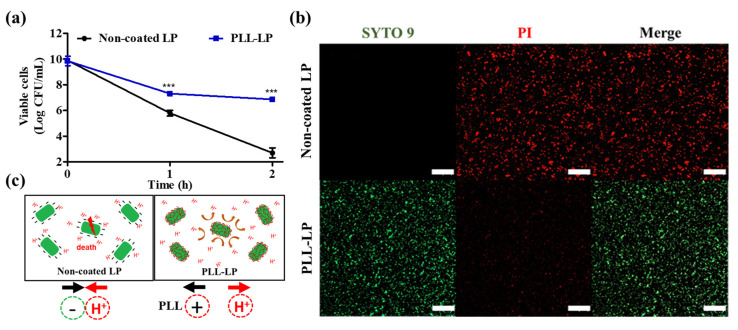
Viability of probiotics in an acidic solution. (**a**) Viable cells after 1 and 2 h of incubation in a pH 2 solution. CFU: colony forming units. The error bar represents the standard deviation (*n* = 8). “***” indicates *p* < 0.001 compared with non-coated LP. (**b**) Confocal laser scanning micrographs of non-coated *L. plantarum* (LP) and poly-L-lysine (PLL)-coated LP (PLL-LP) after 2 h of incubation in a pH 2 solution. Green fluorescence (SYTO 9) represents the intact membrane of live probiotic cells and red fluorescence (PI) represents dead cells with disrupted membranes. The scale bar represents 40 µm. (**c**) Proposed mechanism of the enhanced viability of *L. plantarum* conferred by the PLL coating.

Here, we examined the viability of non-coated and PLL-coated *L. plantarum* in a solution of pH 2. Figure 4a clearly indicates the protective effect of a single-layer PLL coating on the surface of *L. plantarum* incubated in an acidic solution. After 1 and 2 h of incubation in the solution of pH 2, non-coated LP showed a significant reduction in viability (of more than 4 log CFU and 7 log CFU, after 1 and 2 h, respectively). Conversely, the viability of PLL-LP was reduced by only 2 log CFU and 3 log CFU after 1 and 2 h of incubation in a solution of pH 2, respectively.

In addition, confocal microscopy images of non-coated LP and PLL-LP after incubation in pH 2 solution for 2 h are shown in Figure 4b. The green (SYTO 9) and red (PI) fluorescent signals represent live and dead cells, respectively. According to the confocal images, after 2 h of incubation in the pH 2 solution, only dead cells (strong red fluorescence) were observed in the non-coated LP group, whereas the PLL-LP group exhibited a higher number of live cells (green fluorescence) than dead cells. This finding indicated that the viability of PLL-LP was less influenced than that of the non-coated LP by the pH 2 solution. We suggested that the PLL layer, adsorbed as a result of the electrostatic interactions on the surface of the probiotic cells, enhanced the cell membrane’s integrity and limited the diffusion of H+ ions into the probiotic cells. In addition, the change in membrane potential of PLL-LP might have resulted in less adhesion of H+ ions near the probiotic cells than the non-coated, negatively charged probiotic cells. According to the results, PLL-LP exhibited significant advantages in surviving acidic pH conditions when compared to the non-coated LP under identical conditions.

### 3.5. Freeze-Drying and Storage Survivability

**Figure 5:**
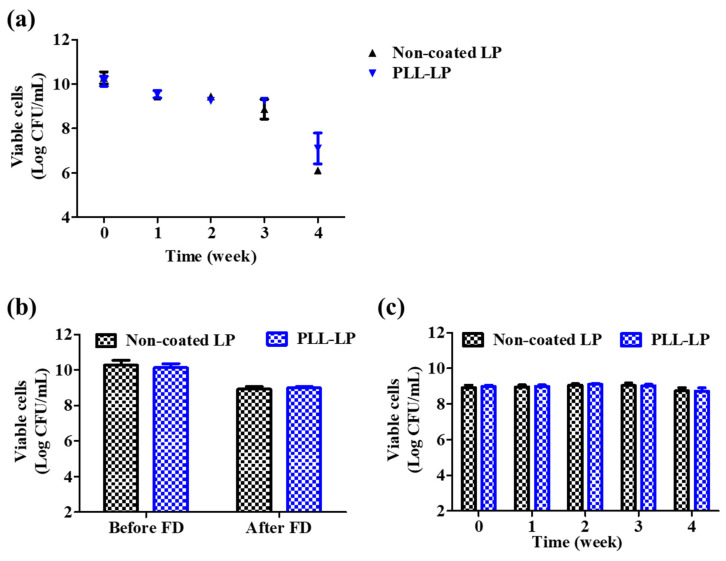
Viability loss of non-coated *L. plantarum* (non-coated LP) and PLL-coated *L. plantarum* (PLL-LP) after freeze-drying and storing (at 4 °C and −20 °C). (**a**) Freshly prepared cells stored at 4 °C for 4 weeks. (**b**) Before and after freeze-drying. (**c**) Freeze-dried cells stored at −20 °C for 4 weeks. The error bar represents the standard deviation (*n* = 8).

As shown in Figure 5a, after 1 week of storage at 4 °C, the viability of PLL-LP decreased from 10.13 ± 0.27 log CFU/mL to 9.54 ± 0.05 log CFU/mL, and that of non-coated LP similarly decreased from 10.28 ± 0.23 log CFU/mL to 9.45 ± 0.16 log CFU/mL. After the fourth week, the viability of non-coated LP decreased to 6.11 ± 0.05 log CFU/mL, whereas that of PLL-LP decreased to 7.10 ± 0.6 log CFU/mL. This may be due to the presence of dissolved oxygen and moisture, which are harmful to the survival and growth of anaerobic species like probiotics [28]. In comparison with non-coated LP, PLL-LP showed a 1 log CFU/mL increase in viability after storage for 1–4 weeks at 4 °C. According to this result, we concluded that the loss in viability of *L. plantarum* after storage at 4 °C was not related to the presence of the PLL coating.

To verify the protective effect of the PLL coating on the probiotic cell membrane, PLL-LP was lyophilized. The survival rate of PLL-LP was determined before and after the freeze-drying procedure and compared to that of non-coated LP. According to the results, as shown in Figure 5b, a decrease in the survival rate of PLL-LP (from 10.13 ± 0.23 log CFU/mL to 8.99 ± 0.07 log CFU/mL) was observed after the freeze-drying process, and the viability of non-coated LPs also decreased (from 10.28 ± 0.27 log CFU/mL to 8.92 ± 0.14 log CFU/mL). The freeze-drying process might inflict a number of stresses on the probiotics, including water crystallization, protein denaturation, low temperatures and membrane injury, which in turn decrease the viability of the probiotics [29,30]. Therefore, we concluded that the PLL coating did not confer any beneficial effects to the probiotic cells under freeze-drying conditions. In addition, the stability of freeze-dried *L. plantarum* at −20 °C was evaluated for 1 month. As shown in Figure 5c, neither the viability of freeze-dried, non-coated LP, nor that of freeze-dried PLL-LP, decreased after 1 month of storage at −20 °C.

### 3.6. In Vivo Viability

**Figure 6:**
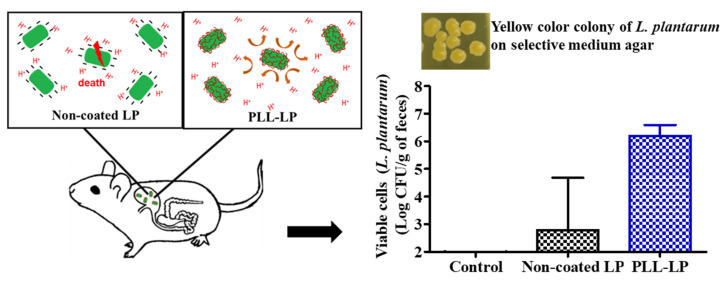
Survival of *L*. *plantarum* after 24 h observed in the feces of ICR mice administered non-coated LP or PLL-LP by oral gavage. The control group comprised ICR mice administered distilled water by oral gavage. The error bar represents the standard deviation (*n* = 4).

To determine the actual survivability of PLL-LP, animal studies have been performed using ICR mice. The survival of PLL-LP in vivo study was performed via administering with the same amount of non-coated and PLL-coated *L. plantarum* via oral gavage. The feces of mice that received PLL-LP by oral gavage contained more colonies of *L. plantarum* (about 6.12 ± 0.4 log CFU/g of feces). Conversely, non-coated LP-treated mouse feces exhibited colony sizes of only 3.79 ± 2.5 log CFU/g of feces, and as expected, there were no *L. plantarum* colonies detected in the feces of the control group. The viability of lactic acid bacteria varies greatly among different people, and even among samples collected from the same subject [31,32]. In addition, the pH value of the upper GIT can vary from 1.7 to 6.1, depending on the fasting interval [33,34]. According to the variances in the physiological mechanisms of an individual, the viability of non-coated bacteria might also vary as the bacteria passes through the GIT. As shown in Figure 6, the standard deviation of probiotic viability in the non-coated LP group was high, which may have been due to the variances in the gastric conditions of each individual mouse. However, the probiotics in the PLL-LP-treated group showed an enhanced viability with a lower standard deviation. Therefore, we concluded that the PLL-LP survived various degrees of gastric acidity and colonized in the intestines with less variation in viability, and can thus be applied in our future studies, such as those concerning the prevention and treatment of inflammatory bowel disease.

## 4. Conclusions

In this study, we significantly enhanced the in vitro viability of *L. plantarum* in acidic conditions by conducting a simple and brief one-step coating process with poly-L-lysine, which produced a coating capable of maintaining the growth and proliferation ability of probiotic cells. In addition, the PLL coating did not negatively affect the viability of *L. plantarum* after it was freeze-dried and stored. It was also determined that the coating enhanced not only the in vitro viability of *L. plantarum* in a gastric acid solution, but also in vivo viability, with little variation. Our results indicated that coating with PLL may be a potentially effective tool for improving the viability of *L. plantarum*. Our study presented a promising approach to designing a probiotic delivery system in the food and pharmaceutical industries.
